# Performance of a three-level triage scale in live triage encounters in an emergency department in Hong Kong

**DOI:** 10.1186/s12245-020-00288-8

**Published:** 2020-06-10

**Authors:** Rex Pui Kin Lam, Shing Lam Kwok, Vi Ka Chaang, Lujie Chen, Eric Ho Yin Lau, Kin Ling Chan

**Affiliations:** 1 24-hour Outpatient and Emergency Department, Gleneagles Hong Kong Hospital, 1 Nam Fung Path, Wong Chuk Hang, Hong Kong Special Administrative Region, China; 2 Emergency Medicine Unit, Li Ka Shing Faculty of Medicine, The University of Hong Kong, Room 514, 5/F, William MW Mong Block, 21 Sassoon Road, Pokfulam, Hong Kong Special Administrative Region, China; 3 Present address: 24-hour Urgent Care Center, Tseun Wan Adventist Hospital, 199 Tseun King Circuit, Tseun Wan New Territories, Hong Kong; 4 School of Public Health, Li Ka Shing Faculty of Medicine, The University of Hong Kong, 2/F, Patrick Mansion Building, 7 Sassoon Road, Pokfulam, Hong Kong Special Administrative Region, China

**Keywords:** Emergency department, Triage, Reliability and validity, Cross-sectional study

## Abstract

**Background:**

Despite its continued use in many low-volume emergency departments (EDs), 3-level triage systems have not been extensively studied, especially on live triage cases. We have modified from the Australasian Triage Scale and developed a 3-level triage scale, and sought to evaluate its validity, reliability, and over- and under-triage rates in real patient encounters in our setting.

**Method:**

This was a cross-sectional study in a single ED with 24,000 attendances per year. At triage, each patient was simultaneously assessed by a triage nurse, an adjudicator (the “criterion standard”), and a study nurse independently. Predictive validity was determined by comparing clinical outcomes, such as hospitalization, across triage levels. The discriminating performance of the triage tool in identifying patients requiring earlier medical attention was determined. Inter-observer reliability between the triage nurse and criterion standard, and across providers were determined using kappa statistics.

**Results:**

In total, 453 triage ratings of 151 triage cases, involving 17 ED triage nurses and 57 nurse pairs, were analysed. The proportion of hospital admission significantly increased with a higher triage rating. The performance of the scale in identifying patients requiring earlier medical attention was as follows: sensitivity, 68.2% (95% CI 45.1–86.1%); specificity, 99.2% (95% CI 95.8–100%); positive predictive value, 93.8% (95% CI 67.6–99.1%); and negative predictive value, 94.8% (95% CI 90.8–97.1%). The over-triage and under-triage rates were 0.7% and 4.6%, respectively. Agreement between the triage nurse and criterion standard was substantial (quadratic-weighted kappa = 0.76, 95% CI, 0.60–0.92, *p* < 0.001), so was the agreement across nurses (quadratic-weighted kappa = 0.81, 95% CI 0.65–0.97, *p* < 0.001).

**Conclusions:**

The 3-level triage system appears to have good validity and reasonable reliability in a low-volume ED setting. Further studies comparing 3-level and prevailing 5-level triage scales in live triage encounters and different ED settings are warranted.

## Background

Triage is the key process in prioritizing care based on urgency. An accurate and reliable triage tool ensures patient safety, upholds clinical justice, improves system efficiency, and reflects ED case-mix and workload [1]. Worldwide, different triage systems are used to fulfil ED operational needs. Currently, 5-level triage systems, including the Australasian Triage Scale (ATS) [2], Canadian Triage and Acuity Scale (CTAS) [3], Manchester Triage System (MTS) [4], and Emergency Severity Index (ESI) [5], are the most studied and widely adopted in developed countries [6, 7]. However, previous studies on these triage systems vary considerably in study design and outcome measurements [8]. Also, there is a lack of strong scientific evidence to support their reliability and predictability of patient outcome [9].

Despite the continued use of 3-level triage systems in many low-volume EDs (annual census < 25,000) in the USA, studies on 3-level triage systems have been lacking compared with the prevailing 5-level triage systems [10]. In 2005, the joint American College of Emergency Physicians (ACEP)/Emergency Nurses Association (ENA) Task Force recommended a move from 3-level triage to 5-level [11], based on two earlier studies that showed inconsistency [12] and a lower reliability of 3-level triage compared with the 5-level ESI [13]. However, a recent study on Turkey’s Ministry of Health’s mandatory 3-level triage instrument, which was modified from the ATS, demonstrated substantial reliability and significant validity [14]. Of note, all these studies were limited by either using small number of paper scenarios [12] or comparing triage systems retrospectively [13, 14], which lack the cues and complexity of the “live” patient presentation [1]. It is worth evaluating 3-level triage system in live triage encounters to better reflect its performance in a real ED setting.

In Hong Kong, all the government-funded public emergency departments under the Hospital Authority (HA) adopt a 5-level triage system based on the Hong Kong Accident and Emergency Triage Guidelines (HKAETG) [15]. The assigned triage category is based on the nurse’s global assessment of the patient’s chief complaint and vital signs. The validity and reliability of the HKAETG 5-level triage system have been found to be satisfactory in a public university tertiary ED [16]. Yet, its applicability in private EDs is uncertain because of different case-mix. In private EDs, the majority of the patients are self-referred and ambulatory [17, 18] corresponding to the triage level 4 (semi-urgent) to 5 (non-urgent) in the public EDs, for which the HKAETG triage system has a lower interrater reliability [16]. Furthermore, differentiation between triage level 4 and 5 is not necessary in private EDs because the waiting time is generally much shorter. To simplify the triage process, our department has introduced a structured 3-level triage system, the Hong Kong 3-level Triage Scale (HK3TS), based on the ATS and HKAETG 5-level triage scale. Similar to ATS, an extensive list of clinical descriptors is used to guide triage for each level [2]. Fractile response time and respective performance thresholds are set for different triage category for service audit (Supplementary Table 1).

In this study, we sought to evaluate the performance of the HK3TS on real patients by studying its validity, reliability, and over- and under-triage rates in our setting.

## Methods

This was a single-centre cross-sectional observational study on the performance of the 3-level HK3TS on actual patients in the 24-hour Outpatient and Emergency Department of Gleneagles Hong Kong Hospital (GHK ED) from 1 May to 1 June 2019. The study was approved by the Research Ethics Committee of GHK (CREC_2019-02). Informed written consent was obtained from both the patients and staff participants.

### Setting and population

GHK ED is a private tertiary ED affiliated with The University of Hong Kong (HKU) Health System. It has commenced its operation since March 2017 with 24 h service started since December 2017. It offers full spectrum of emergency care to patients of all ages. It is staffed 24 h a day by emergency medicine specialists, resident doctors, and registered and enrolled ED nurses. It has around-the-clock access to laboratory services, imaging studies, consultation service by specialists of other disciplines, in-patient beds, and the intensive care unit (ICU). At the time of the study, the annual census was 24,000. Although the GHK ED does not receive patients directly from ambulances, patients with time-critical emergencies, such as acute myocardial infarction, present to GHK ED by own transportation from time to time.

The triage in this ED is performed by ED nurses after patient registration and infection control screen at reception. The duty triage nurse assesses patients in a designated triage room, enters information into the hospital electronic health record system, and assigns a triage category based on global assessment of the patient’s chief complaint and vital signs. The triage scale consists of 3 levels: category 1 (immediate), category 2 (urgent), and category 3 (non-urgent). All ED nurses who undertook triage duty in this study had received in-service training on the use of HK3TS. Some of them had previous work experience with the HKAETG 5-level system in public EDs. After triage, patients are directed to the different care areas, such as cubicle beds or the waiting hall, according to the triage category. For patients who require life-saving interventions (category 1), they are directed to the resuscitation room straight away with emergency medicine specialists summoned immediately.

During the 4-week study period, a convenient sample of GHK ED patients was invited to participate in the study at triage. Patient recruitment was based on the availability of the adjudicator and study nurse, but not on the age or characteristics of the patients. All GHK ED nurses were invited to participate. After obtaining written informed consent from the patients and staff, patients went through triage by the duty triage nurse as usual in the presence of a nurse adjudicator and another study nurse. The adjudicator was a nurse manager who had more than 20 years of clinical experience in emergency medicine. He refined the HK3TS and provided training to ED nurses in our department. His triage rating was regarded as the “criterion standard”. The study nurse was an ED nurse randomly selected from the rest of the team of the same shift. Both of them were refrained from asking questions, giving any clues or hints, or providing any suggestions to the duty triage nurse when they assessed the patient simultaneously. The duty triage nurse entered the patient data and triage rating into the hospital electronic health record system as usual while the nurse adjudicator and study nurse entered their triage ratings in the study data collection forms, which were collected immediately after triage. All of them were blinded to each other’s triage ratings.

### Measurements

As for the validity, it refers to the degree with which the measured acuity level reflects the patient’s true urgency of care needs at the time of triage [19]. No gold standard exists for the evaluation of the validity of triage systems. Predictive validity is the most frequently used method [1, 6, 19]. We assessed the validity of HK3TS by multiple methods. First, the predictive validity was evaluated by studying the proportion of patients requiring hospitalization, transfer to public EDs, referral to other private hospital for admission, ICU admission, and who died in the episode at different triage levels, which are surrogate outcome markers of patient acuity. However, these outcomes may be confounded by factors after the time of triage assessment [1, 19]. Therefore, we also measured the correlation between the triage level and the number of ED interventions required. Each of the imaging studies, laboratory test orders, consultations, and procedures carried out in the ED was equally weighted as one [14]. Furthermore, the ability of HK3TS in identifying patients who required earlier medical attention, i.e. category 1 and 2 cases based on the “criterion standard” of the adjudicator, was determined by the measure of sensitivity, specificity, positive predictive value (PPV), and negative predictive value (NPV).

The reliability of HKTS was assessed by comparing the triage ratings of the duty triage nurse and those by the adjudicator (criterion standard) and study nurse using kappa statistics. The over-triage rate was measured by dividing the number of patients being triaged of a higher level than that given by the adjudicator by the total number of patients recruited. Similarly, the under-triage rate was determined by dividing the number patients being triaged of a lower level than that given by the adjudicator by the total number of patients recruited.

We collected the patient demographic data and data on chief complaints, progress, and outcome using a standardized data collection form. All patient participants were assigned a study code after obtaining informed consent, and data were analysed anonymously.

### Statistics

Missing values were not imputed. Patients with a missing triage rating by any participating nurses were excluded from the analysis. We used descriptive statistics to analyse the distribution of characteristics of the study population and patient outcome. Categorical variables were reported as proportions, and continuous variables as mean ± standard deviation or median with interquartile range (IQR), as appropriate. Chi-square test or Fisher’s exact test, where appropriate, was used to compare the proportion of patient outcomes at different triage levels. Spearman correlation was used to assess the correlation between triage level and the number of ED interventions. The sensitivity, specificity, PPV, and NPV of HK3TS in identifying patients requiring earlier medical attention were calculated with 95% confidence interval (CI) reported.

Reliability was reported as kappa with 95% CI. Unweighted kappa scores reflect exact agreement and treat all disagreements equally. Quadratic-weighted kappa takes into account the level of disagreement and assigns partial credit to closer disagreement, yielding a higher value than unweighted kappa [20]. It is noteworthy that disagreement by more than one triage level is less likely in 3-level triage system than in 5-level system. Yet, weighted kappa is reported in the majority of published triage studies [8]. In this study, both unweighted and quadratic-weighted kappa were reported to facilitate benchmarking with other studies. We interpreted the strength of agreement for the kappa coefficient as ≤ 0 = poor, 0.01–0.2 = slight, 0.21–0.40 = fair, 0.41–0.6 = moderate, 0.61–0.8 = substantial, and 0.81–1 = almost perfect, as proposed by Landis and Koch [21].

We used R statistics (R Foundation for Statistical Computing, Vienna, Austria) to calculate the sample size based on the degree of agreement between the triage nurse and criterion standard. The value for the kappa coefficient to be solely due to chance is assumed to 0.3 (*K*_0_) [22]. According to the literature, the kappa coefficient of the validity of the 5-level HKAETG triage system was 0.77 [16]. We had performed a pilot retrospective study on 100 randomly selected GHK ED patients, which showed a kappa coefficient of 0.76 in the agreement between the triage nurse and criterion standard. According to the patient case-mix in GHK ED, the proportions of category 1, 2, and 3 cases were approximately 1%, 4%, and 95%, respectively. With two raters, an alpha value of 0.05, and the lower bound of kappa at 0.5, the minimum sample size was 141 [23]. To account for a potential 10% loss of recruited cases due to missing values or lost to follow-up, the final sample size was determined as 155. The Statistical Package for the Social Sciences (SPSS) for Windows version 26.0 (IBM Corp., Armonk, NY, USA) was used for data analysis. A two-tailed *p* value < 0.05 was considered statistically significant.

## Results

In total, 154 patients agreed to participate in the study during the study period. One category 1 patient with shock refused to participate. Triage was performed with HK3TS by 17 ED triage nurses, and the study involved 57 different pairs of duty and study nurses. Three patients were excluded because of missing value in triage ratings. We analysed 453 triage ratings of 151 patients. The mean age of the patients was 33.3 years (range 0.75–94.0 years). The demographic and clinical characteristics of the recruited patients are shown in Table 1. There was no category 1 case recruited during the study period. No patients required ICU admission or died.

**Table 1:** Characteristics of the study cohort (*n* = 151)

Characteristics	Number (%)
Age	
≥ 18 years	104 (68.9)
< 18 years	47 (31.1)
Gender	
Male	60 (39.7)
Female	91 (60.3)
ED triage category	
Cat 1	0 (0)
Cat 2	22 (14.6)
Cat 3	129 (85.4)
Trauma	
Yes	25 (16.6)
No	126 (83.4)
Presenting complaints by organ system	
Cardiovascular	6 (4.0)
Respiratory	45 (29.8)
Neurological	10 (6.6)
Gastrointestinal	22 (14.6)
Musculoskeletal	25 (16.6)
Others	43 (28.5)
ED resource utilization	
Imaging	18 (11.9)
Administration of medication	16 (10.6)
Other procedure	29 (19.2)
Outcome	
Treat and discharge	125 (82.7)
Hospital admission	17 (11.3)
Transfer to public hospital ED	7 (4.6)
Referral to other private hospital for admission	5 (3.3)
ICU admission	0 (0)
Episode death	0 (0)

*ED* emergency department; *ICU* intensive care unit

Regarding the predictive validity, the proportions of patients who required hospital admission and referral to other private hospitals for admission significantly increased with a higher triage rating (Table 2). The proportions of patients who required transfer to public EDs were also higher with a higher triage rating, but the difference did not reach statistical significance. Since no patient required ICU admission or died in our cohort, we could not compare the proportion of patients requiring ICU admission or death across different triage levels. The triage level was significantly associated with the number of interventions carried out in the ED (Spearman’s *r* = − 0.40, *p* < 0.001).

**Table 2:** Outcome validity of the 3-level triage system

	Cat 1 (*n* = 0)	Cat 2 (*n* = 22)	Cat 3 (*n* = 129)	*p* value
Hospital admission (%)	N/A	10 (45.5)	7 (5.4)	< 0.001*
Transfer to public hospital EDs (%)	N/A	3 (13.6)	4 (3.1)	0.06*
Refer to other private hospital (%)	N/A	4 (18.2)	1 (0.8)	0.002*
ICU admission (%)	N/A	0 (0)	0 (0)	N/A
Death (%)	N/A	0 (0)	0 (0)	N/A

*ED* emergency department, *ICU* intensive care unit, *N/A* not applicable

*Fisher’s exact test

As for the performance of the 3-level triage system in identifying patients requiring earlier medical attention, the sensitivity was 68.2% (95% CI 45.1–86.1%), specificity 99.2% (95% CI 95.8–100%), PPV 93.8% (95% CI 67.6–99.1%), and NPV 94.8% (95% CI 90.8–97.1%). The inter-observer agreements between the duty triage nurse and the criterion standard and across providers were substantial with an unweighted kappa 0.76 (*p* < 0.001, 95% CI 0.60–0.92) and 0.81 (*p* < 0.001, 95% CI 0.65–0.97), respectively (Tables 3 and 4). Since there was no disagreement of more than one triage level between the raters, the quadratic-weighted kappa values were the same as the unweighted values. The over- and under-triage rates were 0.7% and 4.6%, respectively.

**Table 3:** Criterion validity of the 3-level triage system

		Adjudicator triage rating			
		Cat 1	Cat 2	Cat 3	Total
Duty triage nurse triage rating	Cat 1	0	0	0	0
	Cat 2	0	15	1	16
	Cat 3	0	7	128	135
Total		0	22	129	151

Kappa = 0.76 (*p* < 0.001, 95% CI 0.60–0.92)

**Table 4:** The inter-observer agreement between the duty triage nurse and the study nurse

		Duty triage nurse triage rating			
		Cat 1	Cat 2	Cat 3	Total
Observer nurse triage rating	Cat 1	0	0	0	0
	Cat 2	0	12	4	16
	Cat 3	0	1	134	135
Total		0	13	138	151

Kappa = 0.81 (*p* < 0.001, 95% CI 0.65–0.97)

## Discussion

To the authors’ knowledge, this study was the first study that evaluated the performance of a 3-level triage system in live ED triage encounters. In contrast to previous studies in the USA, our study showed that the HK3TS had an acceptable validity and reliability in a low-volume private ED setting.

Earlier study conducted by Wuerz at el. showed a poor interrater agreement of their 3-level triage system (kappa = 0.35). However, a more detailed review of their study revealed several insufficiencies: there was a lack of clinical descriptor of each triage category and only five scripted patient scenarios were assessed in their study, which did not include obvious emergency patients [12]. Travers et al. compared the validity and reliability of the 3-level system with the 5-level ESI in a university level 1 trauma centre and found that the latter was more effective. However, their study was limited by the retrospective design and comparison of the triage systems at different times, which might be confounded by other time-dependent factors [13]. Our findings are more consistent with the study conducted by Erimşah et al. on Turkey’s Ministry of Health mandatory 3-level triage instrument, which was modified from the ATS and is similar to our 3-level triage scale [14].

It is noteworthy that different research methods might affect the results of triage studies and thus the conclusion drawn. To save costs and manpower, most triage studies, in particular those on 3-level triage systems, used paper scenarios or retrospective chart review [8, 11, 12]. These methods do not capture the visual cues and complex interactions of factors encountered in live triage cases [1]. Worster et al. demonstrated that interrater reliability of CTAS can be quite different in live triage assessments and in paper case scenarios [24]. Considine et al. showed that the addition of visual clues in the form of still photographs delivered by computer resulted in a better interrater reliability in nurse triage using ATS [25]. Studies which lack the important visual clues and dynamic interactions with the patients may underestimate the reliability of triage systems. Our study was conducted in a real triage environment where not only cues (both visual and aural) were available to the raters, but the nurses were also under the pressure of time and stress. We believe this method is more reflective of real-time triage decision-making, and the results add more weight to support 3-level triage.

In the literature, there is no agreed gold standard for the genuine degree of urgency against which the validity of a triage tool can be measured [1]. When surrogate outcome markers were evaluated, a higher triage rating in the HK3TS was significantly associated with a higher proportion of patients requiring hospitalization and referral to other private hospitals for admission. A higher triage rating was also significantly associated with the number of ED interventions required. Since no patient required ICU admission or died in our cohort, we could not use ICU admission or mortality as clinical outcome measures in evaluating predictive validity. Although our triage system was not designed to predict patient ED outcome and the decision on admission may be affected by non-clinical factors, such as bed availability, insurance policy, and financial considerations on the part of the patients, these results support that our 3-level triage system has sufficient discriminative ability in identifying patients who require a higher intensity of care.

In this study, we used the clinical judgement of the adjudicator as the “criterion standard” in determining who required earlier medical attention. Using this approach, the sensitivity of the 3-level HKTS was found to be 68% and the under-triage rate 4.6%. In the literature, the sensitivity of 5-level triage systems in identifying patients requiring life-saving intervention ranges from 77 to 98% [8]. A lower sensitivity in our study can be explained by the difference in evaluation methods (subjective judgement of the adjudicator vs objective record of life-saving intervention). Also, the turnover rate of ED nurses in our department was higher than their counterparts in public EDs (30.7% vs 5.9% [26] in 2018–2019), and many of them have limited ED working experience. Relying on global assessment, which requires knowledge and certain clinical experience, they might not be able to pick up subtle features that would suggest a higher disease acuity during the short patient encounter at triage.

Regarding the reliability, our study showed substantial agreement between the duty triage nurses and the criterion standard (quadratic-weighted kappa 0.76). This figure is higher than that reported by Travers et al. for the 3-level system in the USA (weighted kappa = 0.53) [13], but is comparable with that reported for the 3-level Ministry of Health of Turkey’s mandatory emergency triage instrument (weighted kappa = 0.73) [14]. The respective unweighted and weighted kappa values reported in the literature for the 5-level ATS, MTS, CTAS, and ESI vary considerably and range from 0.43 to 0.84 [25–30] and 0.62 to 0.99 [13, 26, 28–34], respectively. The interobserver agreement across nurses using the HK3TS was almost perfect (quadratic-weighted kappa 0.81). The respective unweighted kappa values for the 5-level ATS, MTS, CTAS, and ESI were 0.40–0.76 [28, 35–37]. The respective weighted kappa values of MTS, CTAS, and ESI were 0.52–0.95 [28, 35, 36, 38–40], respectively.

These findings indicate that the 3-level HKTS is reliable with a consistency comparable with the commonly used 5-level triage systems. Yet the relatively low sensitivity needed to be addressed. The accuracy of triage assessment depends on the triage nurse’s experience, information, and intuition in making the decision, and is inevitably a subjective process [41]. Despite efforts, such as education, triage guidelines, triage algorithms, and audit to reduce the variability of triage assessment, there is little evidence that any of these strategies actually improves the accuracy of triage [1]. In a prospective study on real patients in an urban ED using CTAS, Grafstein et al. demonstrated that a computerized triage menu that linked presenting complaints to preferred triage levels resulted in a high inter-rater reliability [42]. In private EDs where computer systems are used in performing triage, computer aid in decision-making represents a new avenue for future research [43–45].

### Limitations

There were several limitations in this study. First, only a convenience sample was recruited, which might introduce sampling bias. We sought to minimize it by recruiting consecutive patients whenever the adjudicator and study nurses were available during the study period. We have no reason to believe that the patients who presented in their absence had significant different characteristics. Second, the adjudicator and the study nurses could only observe the triage interaction, and they were not allowed to directly question the patients independently. This might affect the accuracy of their triage assessments. Nevertheless, observing a real triage process is much closer to reality than reading paper case scenarios or retrospective chart review, which lacks the visual cues from live interaction. Third, there was no category 1 case in our study. As in many other prospective studies, we had no control on intake of patients to our department. However, our findings were consistent with our pilot retrospective study, which purposely sampled around 10% of category 1 cases. Forth, although the adjudicator was refrained from giving any verbal hints to the duty triage and study nurses, his presence of the adjudicator might lead to a Hawthorn effect during the triage process. Fifth, hospital admission in the private setting may be affected by non-clinical factors, such as insurance cover and bed availability. It might not be a good surrogate of the disease acuity. We sought to overcome this problem by looking into several other indicators. Finally, this was a single-centre study. The findings might not be generalizable to other EDs with different service volume and case-mix.

Despite these limitations, this study provides evidence to support the use of a simplified 3-level triage system in an ED with a relatively low patient volume. Future studies comparing its performance with the prevailing 5-level triage systems in live triage encounters with a multicentre design are warranted.

## Conclusions

When evaluated in live triage encounters, the Hong Kong 3-level Triage Scale appeared to have good validity and reliability in a private ED with a low patient volume. The sensitivity of the scale in identifying patients who require earlier medical attention should be further improved. Further studies comparing 3-level and prevailing 5-level triage scales in live triage encounters and different ED settings are warranted.

## Supplementary information

**Additional file 1:** Supplementary Table 1. The Hong Kong 3-Level Triage Scale

## Data Availability

The datasets generated and/or analysed during the current study are not publicly available because it has not been authorized by the Research Ethics Committee of Gleneagles Hong Kong Hospital.
