# Supplementary material for: Performance of a three-level triage scale in live triage encounters in an emergency department in Hong Kong
Source: Int J Emerg Med. 2020 Jun 10;13:28. doi: 10.1186/s12245-020-00288-8 (PMC7288528; doi:10.1186/s12245-020-00288-8)
Supplement: Supplementary file 1 — Additional file 1: Supplementary Table 1. The Hong Kong 3-Level Triage Scale [file 12245_2020_288_MOESM1_ESM.docx]

Supplementary Table 1. The Hong Kong 3-Level Triage Scale

| Triage category | Description | Performance target |
| --- | --- | --- |
| Category 1 – Critical | - Cardiopulmonary arrest - Signs of upper airway obstruction - Acute respiratory failure (RR>30 or < 10 or SpO_2_ < 90% despite O_2_ therapy) - Shock (SBP<90 mm Hg in adult) - Acute uncontrollable external haemorrhage - Acute deterioration in consciousness with a GCS<13/15 - Extreme tachy- or brady-arrhythmia with a pulse rate<40 or >150 - Chest pain with cold diaphoresis of suspected ACS - Acute onset of stroke related symptoms - Active labour | Immediate medical attention (100%) |
| Category 2 – Urgent | - Respiratory distress requiring O_2_ therapy - Limb emergencies with neurovascular compromise - Severe pain (pain score >7/10) - Acute extremity trauma likely with bony lesion or joint dislocation - Acute retention of urine with palpable bladder - Severe abdominal pain with signs of acute abdomen - Significant loin pain suspicious of renal colic - Pregnancy with per vaginal bleeding and/or abdominal pain - Vertigo/dizziness requiring bed rest - Allergic reaction with features of anaphylaxis or angioedema - Traumatic head injury with a GCS 13-14/15 - Signs of internal bleeding - Active bleeding wound controlled by direct pressure - Hypertension with SBP>220 mm Hg or DBP >120 mm Hg - Hyperpyrexia with a temperature > 40.0 ^o^C - Hypothermia < 35 ^o^C | Medical attention within 15 minutes of triage (95%) |
| Category 3 – Non-urgent | - Other complaints with stable vital signs - Upper respiratory tract infection without high fever - Gastroenteritis symptoms without obvious dehydration - Urinary symptoms with signs of urinary retention or gross haematuria - Mild to moderate joint pain (pain score </=7/10) without gross swelling - Abdominal pain with no signs of acute abdomen - Minor head injury with a GCS 15/15 - Limb injuries without clinical deformities - Simple wounds - Urticarial skin rash with no angioedema - Request for medical check-up or vaccination | Medical attention within 30 minutes of triage (90%) |

Abbreviations: ACS, acute coronary syndrome; GCS, Glasgow Coma Score; SBP, systolic blood pressure; DBP, diastolic blood pressure.
